# Nucleic Acid-Functionalized Gold Nanorods Modulate Inflammation and Dysregulated Intestinal Barriers for Treatment of Ulcerative Colitis

**DOI:** 10.34133/bmr.0195

**Published:** 2025-04-18

**Authors:** Wanghong He, Yanxue Wang, Yifan Zhao, Bingqing Wu, Yilong Chen, Lu Jia, Xinfeng Tan, Yi Liu

**Affiliations:** ^1^ Laboratory of Tissue Regeneration and Immunology and Department of Periodontics, Beijing Key Laboratory of Tooth Regeneration and Function Reconstruction, School of Stomatology, Capital Medical University, Beijing 100070, China.; ^2^ State Key Laboratory of Tribology in Advanced Equipment, Tsinghua University, Beijing 100084, China.

## Abstract

Traditional oral treatments for ulcerative colitis (UC) face marked limitations including their single therapeutic effect, potential off-target interactions, and toxic side effects. In this study, we present nucleic acid-functionalized gold nanorods (NAF AuNRs), a biocompatible nanomaterial designed for the oral treatment of dextran sulfate sodium (DSS)-induced colitis. The NAF AuNRs alleviate immune responses by inhibiting pro-inflammatory macrophages and enhancing the expression of barrier proteins in intestinal epithelial cells. Due to the negatively charged nucleic acid shell, NAF AuNRs preferentially target anionic, inflamed colon tissues upon oral administration, reducing pro-inflammatory cytokine levels and promoting the recovery of intestinal barrier in DSS-induced colitis mice. Collectively, these findings suggest that NAF AuNRs represent an innovative and promising therapeutic approach for UC management, offering novel insights into the application of nucleic acid-functionalized nanomaterials.

## Introduction

Ulcerative colitis (UC) is a chronic, lifelong, and incurable inflammatory intestinal disease affecting about 5 million people worldwide. It severely impacts patients’ quality of life and imposes a considerable medical burden [1]. While the etiology of UC remains unclear, it is commonly linked to intestinal barrier dysfunction, immune dysregulation, and microbial dysbiosis [1,2]. These factors interact to drive UC progression, making it difficult to treat and prone to relapse [3]. Current mainstream treatments, such as oral anti-inflammatory drugs, antibiotics, and immunosuppressants, aim to alleviate inflammation and related symptoms [4,5]. Therefore, their single function limit treatment effectiveness [6]. Moreover, prolonged use of these medications often causes serious complications, including nausea, hypertension, autoimmune disorders, and an increased cancer risk [7]. Consequently, innovative therapeutic strategies are urgently needed to treat UC effectively.

UC features diffuse inflammation and superficial ulceration in the colon mucosa, often accompanied by mucosal barrier defects and microbial dysbiosis [8], where barrier disruption always causes increased intestinal permeability, aggravating infection and inflammation [9]. Thus, 2 critical steps are essential for UC treatment: targeted drug delivery to the inflamed site and activation of multiple therapeutic effects, including restoring the mucosal barriers, suppressing immune responses, and regulating the microbiota. Nanoparticles (NPs) are attractive in drug delivery systems due to their small size, versatility, and stability, which have been applied in treatment of inflammatory bowel disease (IBD) and UC [10–12]. Given that inflamed intestinal mucosa tends to accumulate positively charged proteins, such as transferrin and antimicrobial peptides [10,13], negatively charged NPs can be directed to the inflamed area via electrostatic attraction [14–16]. For example, negatively charged hyaluronic acid has been coated on gold nanoparticle (AuNP)-embedded ceria NPs, promoting NPs to accumulate in the inflamed colon, further reducing inflammation and alleviating injury [15]. Similarly, negatively charged chitosan/alginate polyelectrolytes guided carbon monoxide prodrug-loaded mesoporous polydopamine NPs to the inflamed colon, restoring immune homeostasis and modulating the gut microbiota [16]. However, the synthesis of these NPs involves complex steps, underscoring the need for a simpler NP system for UC therapy.

In the last decade, spherical nucleic acids (SNAs) have gained attention in immunotherapeutic vaccines and tumor immunotherapy modulators, due to high programmability and immunomodulatory properties [17–19]. Typically, SNAs consist of a dense nucleic acid shell and a functional nanomaterial core [19]. Various nucleic acid sequences conjugated to the core form a negatively charged outer layer [17], suggesting possible charge-mediated drug targeting for colitis treatment. Among various NPs, AuNPs are widely used for their ease of modification, high stability, and biocompatibility [20]. Recently, AuNPs modified with 4,6-diamino-2-pyrimidinethiol [21], polyvinylpyrrolidone [22], tannic acid [22], glutathione (GSH) [23], and curcumin [24] exhibit anti-inflammatory effects and regulation capability of intestinal barrier in UC management. However, there is no report on whether AuNPs modified with nucleic acid shell plays anti-inflammatory and barrier recovery effects for UC treatment.

**Fig. 1.**
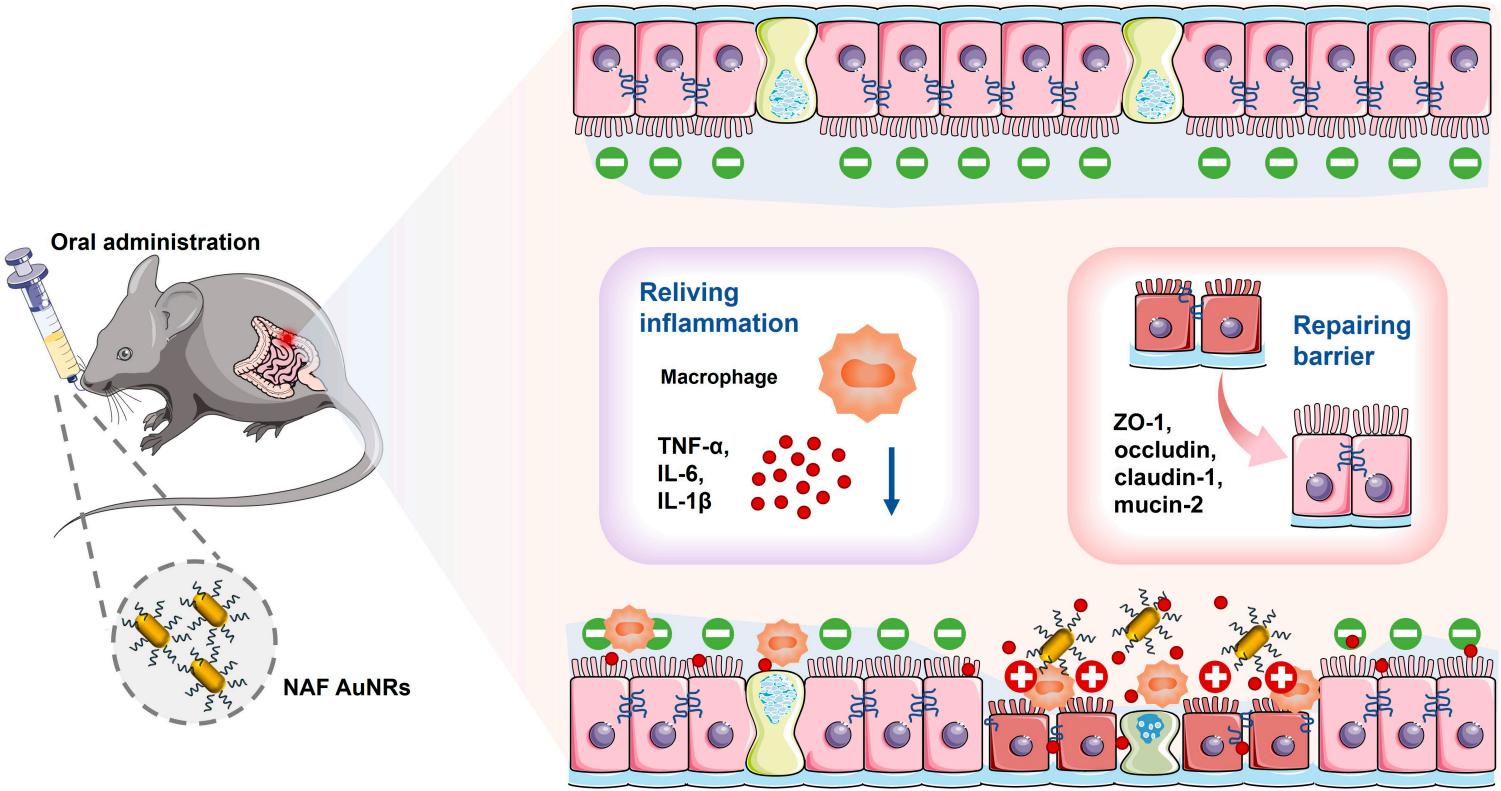
Schematic illustration for the therapeutic mechanism of NAF AuNRs for the targeted treatment of UC.

Herein, we fabricated nucleic acid-functionalized gold nanorods (NAF AuNRs) through a simple and rapid synthetization manner for treating DSS-induced colitis via oral administration. The NAF AuNRs significantly reduced the production of pro-inflammatory cytokines and enhanced the expression of colon barrier proteins both in vitro and vivo. Correspondingly, it effectively alleviated the DSS-induced colitis mice by ameliorating inflammatory responses and restoring the intestinal barrier (Fig. 1). Notably, NAF AuNRs demonstrated excellent compatibility both in vitro and vivo, with minimal effect on major organs. Collectively, these NAF AuNRs hold promise as a potential therapeutic strategy for UC and other inflammatory diseases.

## Materials and Methods

### Materials

NaBH_4_ and AgNO_3_ were purchased by Sinopharm Chemical Reagent Co. Ltd. (China). HAuCl_4_, hexadecyltrimethylammonium bromide (CTAB), phosphate-buffered saline (PBS), and ascorbic acid were from Sigma-Aldrich (USA). *n*-butanol was from MERYER Co. Ltd. (China). SH-T30 DNA probes were synthesized by Sangon Biotech Co. Ltd. (Shanghai, China). CCK-8 Kit and TBE were obtained from Beyotime Biotech Inc. (China). HT-29 cells were from Procell Life Science & Technology Co. Ltd. (China). RPMI 1640 medium and Dulbecco’s modified Eagle’s medium (DMEM) were from Gibco (Thermo Fisher, USA). The artificial gastric fluid, small intestine fluid, and colon fluid were from Beijing OKA Co. Ltd. (China). Anti-ZO-1 antibody, anti-occludin antibody, anti-claudin-1 antibody, anti-mucin-2 antibody, and anti-F4/80 antibody were from Abcam (USA). The mouse interleukin-1β (IL-1β) enzyme-linked immunosorbent assay (ELISA) kits and tumor necrosis factor-α (TNF-α) ELISA kits were purchased from Thermo Fisher (USA). The mouse IL-6 ELISA kits were supplied by BioLegend Inc. (USA). FastPure Complex Tissue/Cell Total RNA Isolation Kits were purchased from Vazyme International LLC (China). PrimeScript RT Reagent Kits were from Takara Bio (Japan). C57BL/6J mice (male) were from SPF Biotechnology Co. Ltd. (China). Animals were housed with 12-h light/dark cycle at 25 °C. All experiments were performed and approved by the Animal Ethical and Welfare Committee in Beijing Stomatological Hospital (approval no. KQYY-202209-003, Beijing, China).

### Synthesis and characterization of the core AuNRs and NAF AuNRs

The core AuNRs were synthesized by a seed-mediated method [25]. To prepare seed solution, CTAB solution (9.75 ml, 0.1 M) was added into HAuCl_4_ solution (0.25 ml, 10 mM) under stirring for 5 min at room temperature. Then, ice-cold NaBH_4_ solution (0.6 ml, 10 mM) was quickly added into the mixture under vigorous stirring for 10 min. Thereafter, the color change from yellow to brown indicated the successful synthesis of Au seed solution. Subsequently, the following solutions were then added sequentially to a 50-ml centrifuge tube: HAuCl_4_ solution (1 ml, 10 mM), CTAB solution (20 ml, 0.1 M), ascorbic acid solution (1.6 ml, 0.1 M), AgNO_3_ solution (0.3 ml, 10 mM), HCl solution (0.2 ml, 0.1 M), and 48 μl of seed solution. Then, the whole mixture was left in the dark for 12 h. The core AuNR solution was collected by centrifugation at 8,000 rpm for 20 min to remove the supernatant and redispersed in 25 ml of deionized water for further use.

NAF AuNRs were synthesized by a butanol extraction method [26]. The core AuNR solution (200 μl) was mixed with SH-T30 DNA probe (5 μl, 100 μM). Then, 1.8 ml of *n*-butanol was added to the mixture with vortex mixing. Subsequently, 400 μl of TBE buffer was added and mixed evenly. The solution was centrifuged at 2,000*g* until liquid phase separation. The particle layer was washed 3 times with 0.01 M PBS by centrifugation at 12,400*g* for 15 min. Finally, the particles were resuspended in 200 μl of 0.01 M PBS buffers.

The morphology, size distribution, zeta potential, and absorption spectrum of the core AuNRs and NAF AuNRs were characterized by transmission electron microscopy (TEM; Thermo Fisher Talos L 120C, USA), Zetasizer Nano ZSE (Malvern, UK), and ultraviolet–visible (UV−vis) spectrophotometer (UV-1900i, Shimadzu, Japan), respectively.

### Cell cytotoxicity assay

The cell viability was investigated by CCK-8 assay. RAW 264.7 cells and HT-29 cells (1 × 10^4^ cells per well) were cultured in 96-well plates in DMEM medium containing 10% fetal bovine serum (FBS) and 1% penicillin-streptomycin liquid (PS) for 24 h under 5% CO_2_ at 37 °C. Then, NAF AuNRs (0.1 M) were added and further incubated with cells for another 24 and 48 h, respectively. After that, cells were treated with CCK-8 (100 μl, 10%) for 1 h. The cell viability was calculated by the ratio of absorbance at 450 nm.

### Intracellular anti-inflammatory effect

Mouse peritoneal primary macrophages were seeded into 12-well plates at a density of 5 × 10^4^ cells per well in RPMI 1640 medium containing 10% FBS and 1% PS. Then, the medium was replaced with fresh medium. The LPS (1 μg/ml) and NAF AuNRs were or were not added into the culture medium. After 24 h of incubation, the cell RNA was extracted by a FastPure Complex Tissue/Cell Total RNA Isolation Kit and prepared for cDNA by PrimeScript RT Reagent Kit. The mRNA gene expression of TNF-α, IL-6, IL-1β, and inducible nitric oxide synthase (iNOS) was analyzed by real-time polymerase chain reaction (RT-PCR) (ABI, USA) and quantified by comparative 2^−ΔΔCt^ method under normalization to glyceraldehyde-3-phosphate dehydrogenase (GAPDH) (primers were synthesized by Sangon Biotech, China, and listed in Table S1). After incubation for 72 h, the cell supernatant was collected and used for inflammatory cytokine detection. The levels of TNF-α, IL-6, and IL-1β were determined by commercial ELISA kits according to the manufacturer’s instruction.

### Protective effect on inflammation-stimulated HT-29 cell

HT-29 cells were seeded into 12-well plates at a density of 5 × 10^4^ cells per well in DMEM containing 10% FBS and 1% PS. Then, the medium was also replaced with fresh medium. The lipopolysaccharide (LPS) (1 μg/ml) and NAF AuNRs were or were not added into the culture medium. After 24 h of incubation, the cell RNA was extracted and prepared for cDNA as before. The mRNA gene expression of mucin-2 and occludin was also analyzed and quantified as before. After incubation for 48 h, the cells were collected and used for assessing protein expression of mucin-2 and occludin by Western blotting (WB). Radioimmunoprecipitation assay (RIPA) lysis buffer (BeiJing Applygen Technologies Inc., China) was used for protein extraction. Then, the extracted proteins were quantified with Bradford assay and boiled after dilution to the same concentration. Next, the samples were separated by sodium dodecyl sulfate–polyacrylamide gel electrophoresis (SDS-PAGE) gels and transferred to polyvinylidene difluoride (PVDF) membranes (Millipore, USA). The membranes were blocked with 5% skim milk in Tris-buffered saline with Tween-20 (TBST) for 1 h at room temperature. Afterward, the membranes were incubated in primary antibody dilutions of ZO-1 [rabbit monoclonal antibody (mAb), dilution ratio: 1:1,000], occludin (rabbit mAb, dilution ratio: 1:1,000), claudin-1 (rabbit mAb, dilution ratio: 1:1,000), and mucin-2 (rabbit mAb, dilution ratio: 1:1,000) overnight at 4 °C. β-Actin (ABclonal, rabbit polyclonal antibody, dilution ratio: 1:5,000) was used for protein loading control. After washing by TBST, the membranes were incubated with secondary antibodies for 1 h at room temperature. Finally, the bands were visualized by a chemiluminescent imager [e-BLOT, e-BLOT Life Science (Shanghai) Co. Ltd., China].

### DSS-induced model of colitis

Following isoflurane inhalation anesthesia (3% induction for 30 s until corneal reflex abolition), mice were subjected to aseptic ear tagging through vertical puncture of the central avascular auricular zone using a pre-sterilized stainless steel ear punch (1.5 mm diameter), with preoperative disinfection performed via concentric 75% ethanol swabbing (10-mm-diameter field). After ear tagging, mice were subjected to adaptive feeding for 7 d.

The mice were randomly divided into 3 groups (*n* = 5 per group) containing the control group, DSS group, and NAF AuNRs group. The control group drunk normal water, while the rest drunk 2.5% DSS solution for 7 d to generate UC. The NAF AuNRs group was orally treated with NAF AuNR solution (50 μl per time) on predetermined days (days 2, 4, and 6). The control and DSS groups only received PBS solution. The body weight, degree of fecal bleeding, and softness of all groups were recorded daily throughout the entire experimental period. On day 8, the colons, major organs including heart, liver, spleen, lung, and kidney, and blood of all the groups were collected for further examination. The disease activity index (DAI) scores were depended on weight loss scores (0, less than 1%; 1, 1% to 5%; 2, 5% to 10%; 3, 10% to 20%; and 4, more than 20%), hematochezia scores (0, negative; 2, positive blood traces in stool; and 4, macroscopic), and stool consistency (0, hard; 2, soft; and 4, diarrhea) [27].

### Assessment of the histological activity index

Colons were fixed in 4% paraformaldehyde for 24 h, then embedded in paraffin, and sliced into 4-μm sections. The sections were stained with hematoxylin and eosin (H&E) to evaluate epithelial morphology and damage. The histological activity index (HAI) scores were summed by crypt gland destruction scores (0, normal; 1, a small amount loss of crypt; 2, a large amount loss of crypt; 3, extensive loss of crypt), goblet cell loss scores (0, normal; 1, a small amount loss of goblet cells; 2, a large amount loss of goblet cells; 3, extensive loss of goblet cells), and inflammatory cell infiltration scores (0, normal; 1, infiltration around crypt glands; 2, infiltration of mucosal muscle layer; 3, widespread infiltration of mucosal muscle layer; 4, submucosal infiltration).

### Analysis of inflammatory cytokines and barrier-related protein expression in the colon

Total RNA was extracted from colons by a FastPure Complex Tissue/Cell Total RNA Isolation Kit and prepared for cDNA by PrimeScript RT Reagent Kit. The mRNA gene expression of TNF-α, IL-6, IL-1β, iNOS, ZO-1, and occludin was analyzed by RT-PCR (ABI, USA), and quantified by comparative 2^−ΔΔCt^ method under normalization to GAPDH (primers were synthesized by Sangon Biotech, China, and listed in Table S1). Colon tissues were lysed and centrifuged, and the supernatant was used for assessing protein expression of ZO-1, claudin-1, occludin, and mucin-2 by WB. The reagents and operating procedures were completely the same as descripted in the “Intracellular anti-inflammatory effect” and “Protective effect on inflammation-stimulated HT-29 cell” sections.

### Microbial community analysis

The collected cecal contents were snap frozen in liquid nitrogen and then stored at −80 °C. Total genomic DNA from samples was extracted using CTAB method. DNA concentration and purity was determined using 1% agarose gels. 16*S* ribosomal RNA (rRNA) genes of distinct regions (16*S* V3–V4) were amplified using specific primers 341F (5′-CCTAYGGGRBGCASCAG-3′) and 806R (5′-GGACTACNNGGGTATCTAAT-3′) with the barcode. After PCR amplification, the products were purified with Universal DNA (TianGen, China). Library construction and sequencing were performed by Novogene (Tianjin, China).

### Histological analysis

Immunohistochemical (IHC) staining was conducted to investigate the expression of IL-1β, IL-6, and TNF-α to compare the inflammatory status of different groups. Subsequently, immunofluorescence (IF) staining for ZO-1, claudin-1, occludin, and mucin-2 was utilized to observe the damage and recovery of colonic mucosal barrier, whereas IF staining for F4/80 marker was utilized to detect macrophage infiltration.

### Biosafety

Major organs including heart, liver, spleen, lung, and kidney were sliced to 4 μm sections and stained with H&E to assess the tissue toxicities.

### Statistical analysis

All analyses were conducted with GraphPad Prism 8.3 with one-way analysis of variance (ANOVA) followed by Tukey post hoc test. Statistical significance was indicated as *P < 0.05, **P < 0.01, ***P < 0.001, ****P < 0.0001. The effect size of η^2^ was calculated by SPSS Statistic 24.

## Results

### Synthesis and characterization of NAF AuNRs

**Fig. 2.**
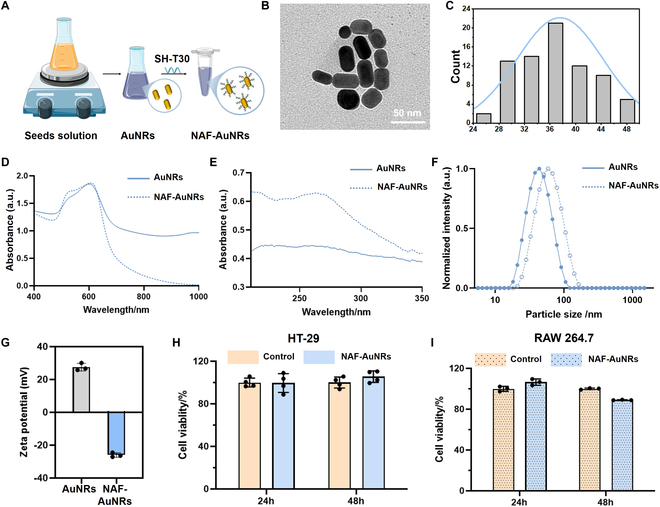
Preparation and characterization of NAF AuNRs. (A) Schematic design of the preparation of NAF AuNRs. (B) TEM image and (C) size distribution of AuNRs. Absorption spectrum of AuNRs and NAF AuNRs at the range from 400 to 1,000 nm (D), and from 210 to 350 nm (E). (F) Dynamic light scattering (DLS) results and (G) zeta potential of the AuNRs and NAF AuNRs. Cell viability of (H) HT-29 cells and (I) RAW 264.7 cells at 24 and 48 h measured by the CCK-8 assay after the NAF AuNR treatment.

According to the previous seed-mediated method and some variations [25], we first synthesized the core AuNRs, which exhibit good dispersibility in aqueous solution with a deep blue-purple color (Fig. 2A and Fig. S1). The TEM images indicated that the core AuNRs display a rod-like morphology with an average length of 37.56 ± 6.75 nm (Fig. 2B and C). Considering the evidence that G-rich SNAs were apt to induce macrophage activation and lead macrophages to produce more inflammatory cytokine (e.g., TNF-α, IL-6, and IL-1β) and nitric oxide (NO) than their poly-T counterpart [28], we chose Poly-T single-stranded oligonucleotides (T30) to form external nucleic acid shell. Combining the thiol (SH)-T30 sequence with the butanol extraction method [26], NAF AuNRs were prepared (Fig. S2), whose dispersibility and color are both similar with the core AuNRs (Fig. 2A and Fig. S1). The UV–vis spectrum showed that the shape of the absorption peak at 520 nm of NAF AuNRs changes compared to the core AuNRs (Fig. 2D), because of the surface plasmon resonance (SPR) [21]. Meanwhile, a new absorption peak appears at 260 nm for NAF AuNRs (Fig. 2E), corresponding to the absorption peak of nucleic acid. The average hydrodynamic sizes of NAF AuNRs are larger than those of the core AuNRs (Fig. 2F). Moreover, the zeta potential of the core AuNRs after conjugation of the thiol (SH)-T30 sequence decreases from 27.5 mV to −26 mV (Fig. 2G). These results all prove the successful preparation of NAF AuNRs, where the core AuNRs are coated with a layer of T30. Next, we assessed the stability of NAF AuNRs in gastrointestinal fluids. The results showed that their particle size gradually increased after more than 6 h of incubation in artificial gastric fluid (Fig. S3), suggesting progressive aggregation. In contrast, NAF AuNRs remained more stable in artificial small intestinal and colonic fluids (Fig. S4), indicating their potential for oral administration.

### Evaluation of cytotoxicity, anti-inflammation, and barrier recovery of NAF AuNRs in vitro

The cytotoxicity of NAF AuNRs was evaluated in vitro by cell viability measurement using HT-29 and RAW 264.7 cells. As shown in Fig. 2H and I, NAF AuNRs exhibit no obvious toxicity in both cells under 24 and 48 h, implying their great cytocompatibility.

**Fig. 3.**
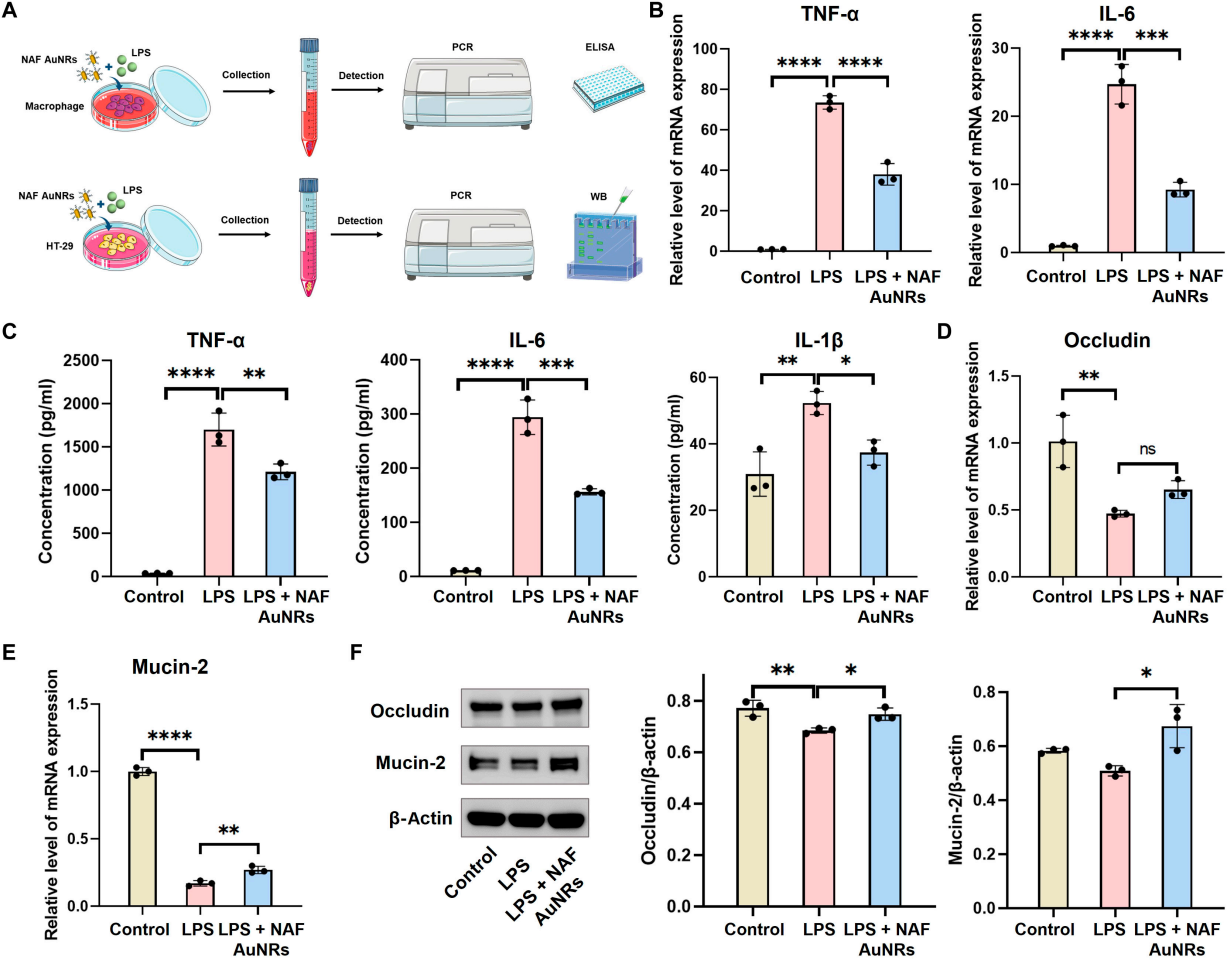
Anti-inflammation and barrier recovery of NAF AuNRs in vitro. (A) Schematic representation of NAF AuNR treatment for the LPS-stimulated mouse peritoneal macrophages and HT-29 cells. (B) Real-time quantitative PCR (RT-qPCR) analysis of the gene expression of TNF-α and IL-6 of LPS-stimulated mouse peritoneal macrophages (effect size of η^2^ = 0.990 and 0.978). (C) ELISA results of pro-inflammatory cytokines including TNF-α, IL-6, and IL-1β from the supernatant of LPS-stimulated mouse peritoneal macrophages (effect size of η^2^ = 0.980, 0.983, and 0.836). RT-qPCR analysis of the gene expression of (D) occludin and (E) mucin-2 of LPS-stimulated HT-29 cells (effect size of η^2^ = 0.840 and 0.997). (F) WB results of occludin and mucin-2 of LPS-stimulated HT-29 cells (effect size of η^2^ = 0.788 and 0.840). *P < 0.05, **P < 0.01, ***P < 0.001, ****P < 0.0001.

In the inflamed intestine, the pro-inflammatory macrophage counts were significantly elevated, and pro-inflammatory factors, such as IL-6, iNOS, IL-1β, and TNF-α, were continuously released, exacerbating the inflammation and destroying the gut tissues [29]. Thus, our study first assesses the ability of NAF AuNRs to suppress the production of pro-inflammatory cytokines in LPS-activated mouse peritoneal-derived macrophages (Fig. 3A). After the stimulation of LPS (1 μg/ml) for 24 h, the gene expression of proinflammatory cytokines (TNF-α, IL-6, and IL-1β) in peritoneal macrophages was significantly up-regulated. Noticeably, the up-regulated mRNA expression of TNF-α, IL-6, IL-1, and iNOS was greatly inhibited by intervention with NAF AuNRs (Fig. 3B and Fig.S5). Consistently, ELISA results also showed that the elevated protein levels of TNF-α, IL-6, and IL-1 after LPS exposure were considerably down-regulated by NAF AuNRs (Fig. 3C), demonstrating the effective anti-inflammatory capacity of NAF AuNRs in LPS-induced mouse peritoneal-derived macrophages.

As the destructive mechanical barrier and mucus barrier are driven by the persistent inflammatory state in the colon, we utilized LPS-stimulated HT-29 colon cells by assessing the changes in the levels of occludin and mucin-2, the 2 main components in the intestinal barrier (Fig. 3A). The HT-29 cells were stimulated with LPS (10 μg/ml) for 24 h with or without NAF AuNR treatment, and RT-PCR and WB were examined for the levels of occludin and mucin-2. The results revealed that LPS exposure reduced the expression of occludin and mucin-2 in HT-29 colon cells both at the mRNA and protein levels, whereas the decrease was significantly reversed by NAF AuNR treatment (Fig. 3D to F). All these results showed that NAF AuNRs could efficiently suppress macrophage-associated inflammation and enhance HT-29 colon cell-mediated barrier function.

### Amelioration of DSS-induced colitis in mice by oral administration of NAF AuNRs

To further investigate whether NAF AuNR intervention is effective in alleviating intestinal inflammation caused by DSS, we established the UC model using DSS. The mice were categorized into 3 groups: control, DSS, and DSS + NAF AuNRs groups.

**Fig. 4.**
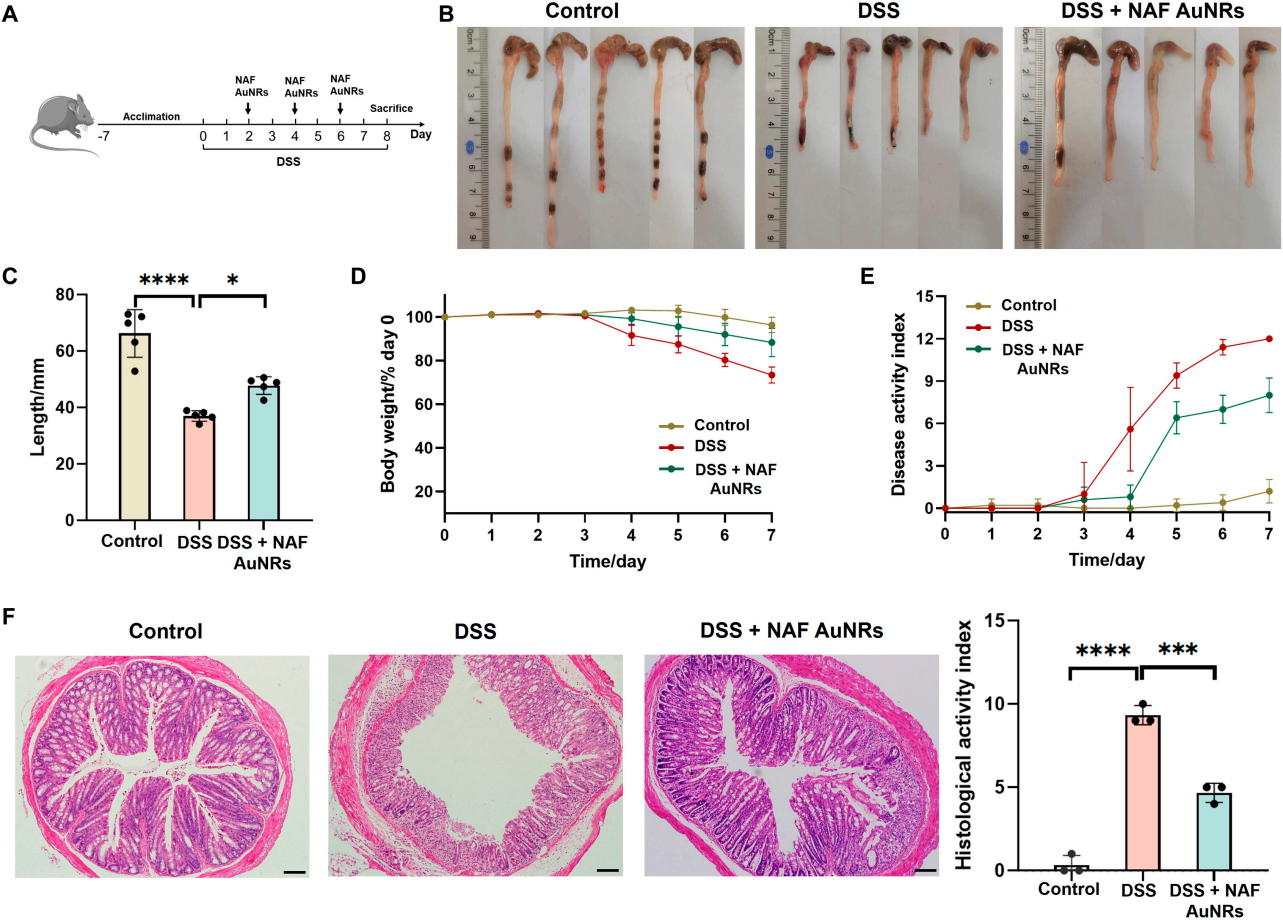
Evaluation of the efficacy of NAF AuNR treatment in the mice with DSS-induced UC. (A) Schematic illustration of the establishment and therapeutic schedule of the DSS-induced UC mice. (B) Macroscopic colon appearance of mice with different treatments. (C) Colon length of mice with different treatments (effect size of η^2^ = 0.866). (D) Body weight and (E) DAI score change in each group for 7 d. (F) Representative images of H&E staining image and HAI of each group (effect size of η^2^ = 0.984). Scale bar, 50 μm. ***P < 0.001, ****P < 0.0001.

As shown in Fig. 4A, 2.5% DSS solution for 7 d was used in mice for establishing UC model, during which NAF AuNRs were given by oral administration every other day for treatment. The histopathological examination in the major organs demonstrated no obvious pathological changes in each group, proving hardly any side effects and toxicities and biosafety of NAF AuNRs in vivo (Fig. S6). Additionally, inductively coupled plasma mass spectrometry (ICP-MS) quantification demonstrated nonsignificant gold deposition in major organ systems (cardiovascular, hepatic, splenic, pulmonary, renal), with localized accumulation restricted to the gastrointestinal tract (Fig. S7). Notably, the 7-d experimental duration constitutes a methodological constraint, necessitating future longitudinal investigations to establish gold kinetic profiles and evaluate potential chronic exposure effects. Then, we examined the changes in colon length, body weight, and DAI.

Due to the mucosal damage, intestinal epithelium barrier disruption, and dehydration in the development of UC, colon shortening is a critical parameter for the evaluation of the severity of inflammation [29]. As shown in Fig. 4B and C, reduced colon length in the DSS-treated groups was observed, while the colon length in the NAF AuNRs group was longer, revealing a significant protective role of NAF AuNRs. Measurement of body weight showed that serious weight loss in the DSS-induced group was rescued following the NAF AuNR treatment (Fig. 4D). In addition, compared to the control group, the score of DAI of the DSS group was higher, demonstrating aggravation of the colitis. In contrast, the score of DAI was reduced following NAF AuNR administration, further proving the curative effect (Fig. 4E). Additionally, a comparative analysis between NAF AuNRs and 5-aminosalicylic acid (5-ASA, mesalamine) demonstrated comparable therapeutic efficacy in ameliorating UC-associated pathophysiological parameters, including colon length preservation, body loss, and DAI (Fig. S8). Altogether, the results above illustrated the therapeutic value of NAF AuNRs against colitis.

To further assess the histopathological changes of the colon tissues and the degree of colonic lesions after NAF AuNR intervention, we performed H&E staining and scored the lesions of colonic tissues based on HAI. As shown in Fig. 4F and Fig. S9, DSS supplementation induced severe intestinal crypt necrosis and the abundant loss of intestinal glands and goblet cells in colon tissue, accompanied by infiltration of lymphocytes and granulocytes. In contrast, these inflammation-associated features were partially relieved, along with a low histological score after NAF AuNR treatment. Similarly, NAF AuNRs demonstrated lower HAI scores compared to 5-ASA treatment, suggesting superior protective properties (Fig. S8). Considering the comparable therapeutic profiles between NAF AuNRs and 5-ASA in colon length preservation, weight loss, and DAI score, extended therapeutic duration trials are required to characterize their therapeutic differentiation. Taken together, the results above indicated that the oral administration of NAF AuNRs is highly effective in treating DSS-induced colitis.

### Anti-inflammatory effects by NAF AuNRs in vivo

**Fig. 5.**
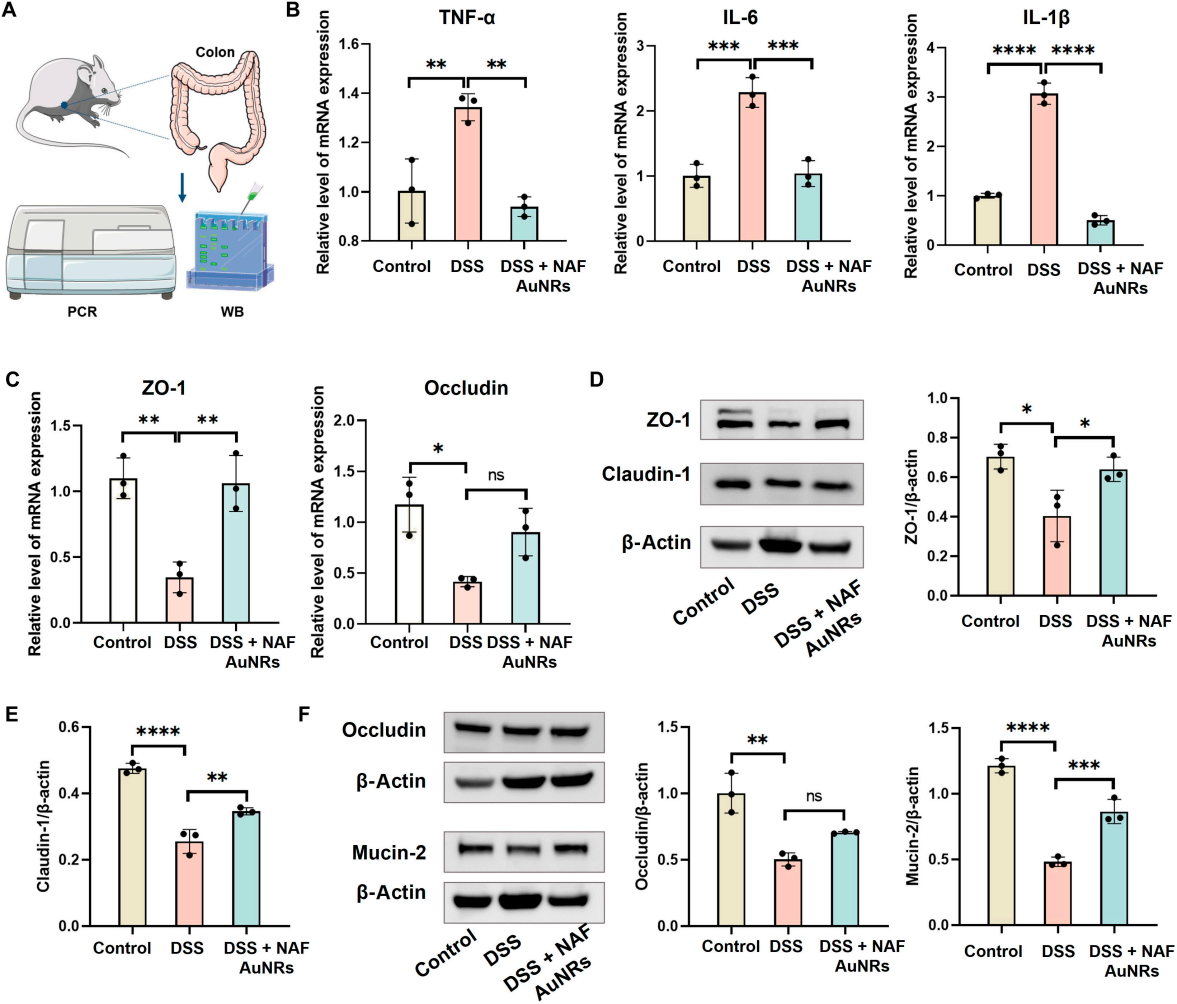
Anti-inflammation efficacy and recovery of impaired intestinal barrier of NAF AuNRs in vivo. (A) Schematic representation of evaluation methods. RT-qPCR analysis of the gene expression of (B) TNF-α and IL-6, and (C) ZO-1 and occludin of colon tissues in each group (effect size of η^2^ = 0.867, 0.928, 0.867, and 0.774). WB results of (D) ZO-1, (E) claudin-1, (F) occludin, and mucin-2 of colon tissues in each group (effect size of η^2^ = 0.753, 0.957, 0.883, and 0.970). *P < 0.05, **P < 0.01, ***P < 0.001, ****P < 0.0001.

**Fig. 6.**
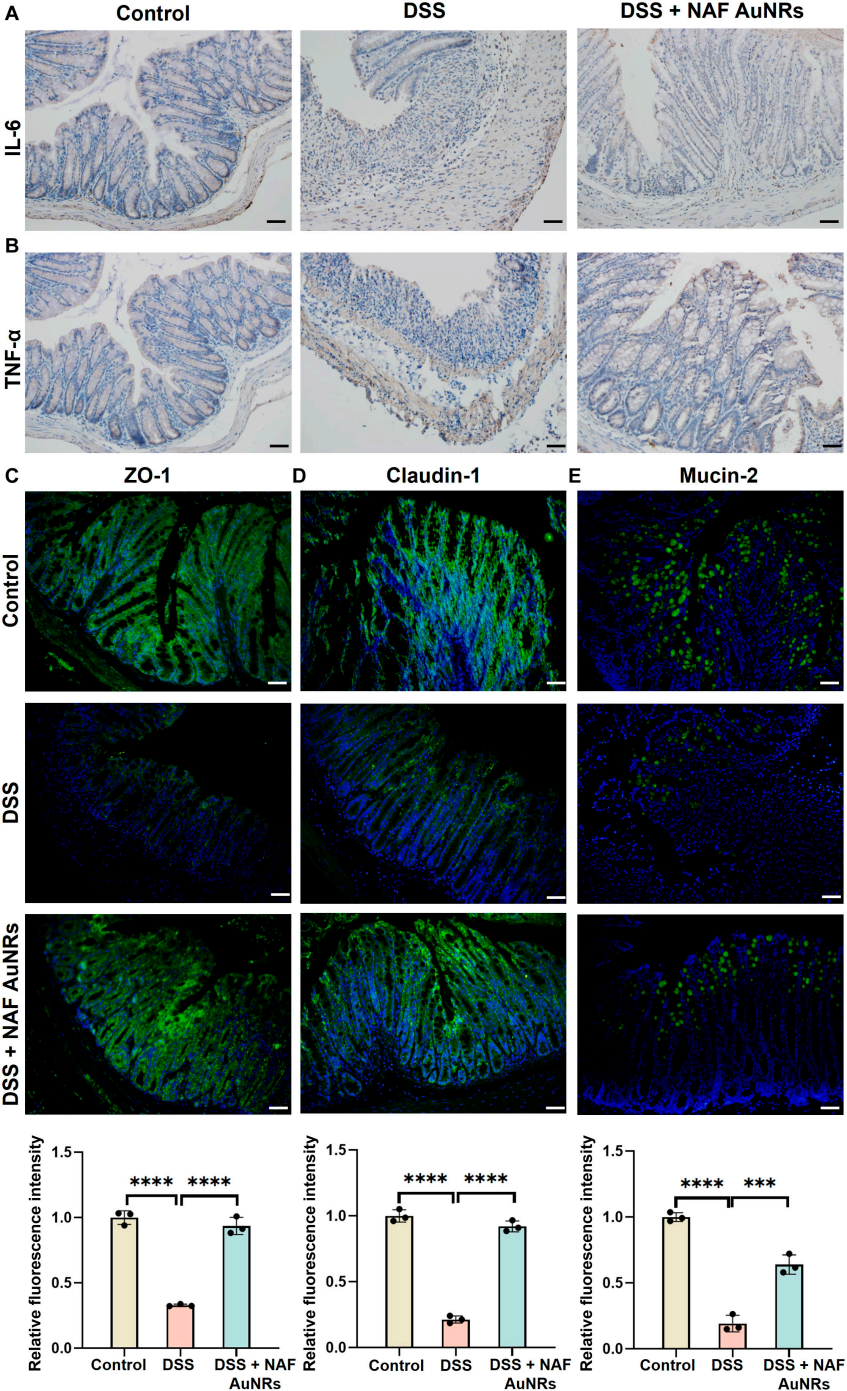
Therapeutic effect evaluation of NAF AuNRs in vivo through histology staining. Representative IHC images for (A) IL-6 and (B) TNF-α in each group. Representative IF staining and quantification of (C) ZO-1, (D) claudin-1, and (E) mucin-2 staining (effect size of η^2^ = 0.983, 0.992, and 0.979). Scale bar, 50 μm. **P < 0.01, ****P < 0.0001.

In the progression of UC, excessive secretion of pro-inflammatory cytokines induces intestinal inflammatory response, leading to aggravated damage. Next, we investigated the therapeutic effects by examining inflammation-related cytokines in the colons at the molecular level (Fig. 5A). Macrophages play an important role in maintaining and regulating intestinal homeostasis; however, in colitis, inflammatory macrophages are the contributing factor in the development of colitis [30]. Therefore, we further analyzed whether NAF AuNRs could regulate macrophage-mediated inflammation by examining macrophage infiltration and inflammatory cytokines from colon. IF revealed that the number of macrophages (F4/80^+^) was increased in the colon of colitis mice compared with expression in the normal mice, which were regulated by NAF AuNRs (Fig. S10). Furthermore, compared with the control group, RT-PCR revealed that there was an obvious increase in the expression of the typical inflammation-related cytokines, mainly including TNF-α, IL-6, IL-1β, and iNOS in the DSS-induced colitis model. In contrast, the levels of these cytokines were remarkably down-regulated following NAF AuNR oral administration (Fig. 5B and Fig. S11). Meanwhile, the IHC assay showed a significant decrease in the levels of TNF-α, IL-6, and IL-1β in colon tissues in the DSS group compared with the control group, which was reversed by NAF AuNR administration (Fig. 6A and B and Fig. S10). Taken together, NAF AuNRs can effectively reduce the expression of these pro-inflammatory cytokines in colitis.

### Repair of gut barrier function by NAF AuNRs in vivo

The tight junction (TJ) proteins are key elements in maintaining the integrity of gut mucosal barrier function. The colitis progression inevitably leads to TJ decrease, which damages the colonic mucosal barrier, thus making it susceptible to bacterial infections and inflammation recurrent [31]. Besides, due to electrostatic interactions, the permeability of claudin protein to positively charged cations is reduced [32], which may enhance the electrostatic interaction between negatively charged NAF AuNRs and TJ, thereby promoting the functionality of NAF AuNRs on the mucosal surface.

To examine the therapeutic effects of NAF AuNRs on epithelial barrier function in colitis, we analyzed the expression of ZO-1, claudin-1, and occludin, the critical components of TJs, by RT-PCR, WB, and IF. As shown in Fig. 5C, the mRNA expression of ZO-1 and occludin was decreased in the DSS model, while oral administration of NAF AuNRs significantly up-regulated the expression of these 2 genes. Besides, the protein expression of ZO-1, claudin-1, and occludin also increased following NAF AuNR administration (Fig. 5D to F). Compared with the DSS group, the IF assay results demonstrated that NAF AuNR treatment exerted a higher level of ZO-1, claudin-1, and occludin, and normalized a more intact structure of the epithelial barrier (Fig. 6C and D and Fig. S12). Besides, mucin-2 is a mucin that reflects the intestinal mucus layer, which is against both commensal microbes and invading pathogens. As illustrated in Fig. 5F, there was a significant decrease in the expression of mucin-2 at the protein level compared to the control group, showing a serious loss of mucus secretion in colon tissues. However, the damage was substantially restored by NAF AuNR administration. Correspondingly, IF assay results displayed that NAF AuNR treatment effectively prompted mucin-2 secretion (Fig. 6E). The results above strongly supported that NAF AuNRs had a therapeutic potential to restore epithelial barrier function mainly by maintaining TJs and mucus secretion in the DSS-induced colitis model. Previous study has shown that a combination of anti-inflammatory drugs and TJs’ regulators effectively treat IBD through repairing barrier function and improving anti-inflammatory efficacy [27]. Similarly, our NAF AuNRs indicated dual function in anti-inflammation and barrier reconstruction in UC treatment, implying their therapeutic potential for the management of IBD.

### Modulation of gut microbiome in colitis mice by NAF AuNRs

Intestinal dysbiosis is a key contributor to inflammation and intestinal damage, driving the progression of IBD [33]. To evaluate the impact of NAF AuNRs on gut microbiota composition in DSS-induced colitis mice, we performed high-throughput 16*S* rRNA gene sequencing on collected stool samples. Venn diagram analysis identified 1,650 operational taxonomic units (OTUs) across the control, DSS, and NAF AuNR treatment groups, with 433 OTUs unique to the DSS group and 353 to the NAF AuNR group. Notably, the overall OTU count decreased significantly after NAF AuNR treatment compared to the DSS group (Fig. S13A), suggesting microbiota modulation. α-Diversity analysis (Simpson and Shannon indices) revealed that DSS administration decreased microbial richness, although no significant differences were observed between the DSS and NAF AuNR groups (Fig. S13B and C). β-Diversity analysis via principal coordinates analysis (PCoA) demonstrated that DSS disrupted the gut microbiota composition, while NAF AuNRs shifted it closer to that of the control group (Fig. S13D). Linear discriminant analysis effect size (LEfSe) analysis identified key microbial taxa enriched in the NAF AuNR group compared to the DSS group (Fig. S13E). At the phylum level, NAF AuNRs significantly increased the relative abundance of *Firmicutes* and *Actinobacteria* (Fig. S13F), which are known to be reduced in UC and linked to disease progression [34]. Overall, these findings demonstrate that in colitis mice, NAF AuNRs altered β-diversity of gut microbiota and increased the abundance of beneficial taxa (predominantly *Firmicutes*) to modulate the gut microenvironment. 

## Discussion

In clinical practice, the primary goal of UC treatment is symptom control. Commonly used therapies include glucocorticoids, immunomodulators, aminosalicylic acid drugs, and biologics. However, the side effects and instability of these treatments in vivo remain significant challenges [35]. Compared to traditional drugs, targeted nano-drug delivery systems offer promising advantages by directly targeting inflammatory sites, thereby increasing local drug concentrations. These systems can also enhance drug solubility, immunogenicity, and stability, potentially improving therapeutic efficacy and representing a novel targeted approach for UC treatment [10,36]. Among these systems, the tunable characteristics of nanoparticles (NPs)—including size, shape, and surface properties—have garnered particular attention, making them a promising new avenue for targeted UC therapy [10,36].

A variety of NP-based drugs have been explored for the targeted therapy of UC, including intravenous hollow CeO_2_ NPs modified with polyethylene glycol (PEG) [37], intravenous PEGylated ceria nanozymes [38], orally administered cyclosporin A-loaded NPs coated with macrophage membranes or leukocyte membranes [35], oral P127-AuS@CURs assisted by near-infrared light [24], and so on. Compared to intravenous and assisted delivery, oral administration is simpler, is more convenient, and generally has better patient compliance [38]. The NAF AuNRs presented in this study enable adjuvant-free oral administration, demonstrating enhanced clinical applicability compared to existing systems such as PEGylated ceria nanozymes that require complex delivery protocols. The reported oral UC-targeted NP drugs include charge-, ligand receptor-, degradation-, and microbiota-mediated targeting systems [10]. Among these approaches, using surface-negative charges for targeting UC sites is a simple yet effective strategy compared to ligand, enzyme, or antimicrobial protein modifications [23,36]. Previous studies have demonstrated that spherical nucleic acids with negatively charged outer layers possess immunomodulatory properties [17–19]. AuNPs functionalized with small molecule drugs have also been shown to effectively reduce inflammation and restore the intestinal mucosal barrier in UC [21–24]. Our study is the first to explore the use of NAF AuNRs for the targeted treatment of UC. Previous studies established that G-rich sequences preferentially induce macrophage pro-inflammatory polarization, as evidenced by significantly elevated secretion of IL-6, IL-1β, and TNF-α, coupled with enhanced surface expression of CD40, CD69, and CD86 relative to poly-T sequences [28]. Guided by these findings, we strategically selected the poly-T sequence (T30) for anti-inflammatory therapeutic development. Both in vitro and in vivo experiments confirmed their dual efficacy in alleviating inflammation and restoring the intestinal barrier. The synthesis processes of charge-targeted NPs reported in previous studies often involve complex multilayer encapsulation or modification procedures [6,15,23,36]. In contrast, the synthesis and preparation process of NAF AuNRs are much simpler. Additionally, in contrast to other NPs (e.g., PEGylated cerium nanozymes and carbon dots) that mediate antioxidant and anti-inflammatory therapeutic effects primarily through reactive oxygen species (ROS) scavenging [39,40], NAF AuNRs exhibit dual therapeutic efficacy via immunomodulatory mechanisms. Specifically, they attenuate inflammation through macrophage functional regulation while enhancing intestinal mucosal barrier repair via modulation of colonic epithelial secretory activity. This distinct mechanism highlights the potential of nucleic acid-functionalized nanomaterials in UC management. However, the long-term stability of NAF AuNRs in gastrointestinal fluids requires further optimization. Future studies will focus on enhancing their stability through PEGylation or protective encapsulation, comparing the in vivo therapeutic effects of different encapsulation strategies, and identifying the optimal stabilization approach to maximize the therapeutic efficacy of NAF AuNRs. Moreover, plasmid-based delivery systems exhibit reduced immunogenicity and enhanced biosafety profiles as nucleic acid carrier, but they suffer from rapid enzymatic degradation and inefficient therapeutic delivery [41,42]. In contrast, a steric hindrance effect from densely packed surface nucleic acids confers enhanced NAF AuNR resistance to nuclease-mediated degradation [43,44]. Meanwhile, the 3-dimensional (3D) spatial organization of oligonucleotides promotes endocytosis, enabling efficient therapeutic delivery [44,45]. This study demonstrates for the first time that nucleic acid-modified gold nanorods can alleviate DSS-induced colitis by modulating inflammation and restoring the intestinal barrier. Our findings not only expand the understanding of the function of nucleic acid-modified nanomaterials but also suggest potential new avenues for their therapeutic application.

Inflammation and intestinal barrier dysfunction are central mechanisms in the pathogenesis of UC and key targets for the development of prevention and treatment drugs for the disease [46]. The crosstalk between intestinal barrier and local immune cells plays a critical role in UC progression. Dysregulated immune responses can disrupt intestinal homeostasis, triggering inflammatory responses. In our study, we found that NAF AuNR treatment markedly inhibited the inflammatory response in both DSS-induced colitis mice and LPS-induced macrophages. The intestinal barrier serves as the first line of defense against toxins and antigens, and epithelial dysfunction is a hallmark of UC [46]. TJ proteins, including occludin, claudin, and ZO-1, are critical components of the intestinal physical barrier and are essential for maintaining intestinal homeostasis and regulating permeability [47]. Additionally, the mucus barrier plays a vital role in preserving the integrity of the intestinal barrier, with secretory mucin (mucin-2) being the primary component of this barrier [48]. Under the conditions of UC, reduced levels of TJ proteins and mucin-2 can destroy intestinal barrier function and exacerbate intestinal inflammation. In the present study, we observed significantly lower expression of TJ proteins and mucin-2 in DSS-induced colitis mice compared to normal mice, indicating intestinal barrier disruption. However, the above indicators were remarkably reversed following NAF AuNR administration. These findings suggest that the protective effects of NAF AuNRs on ameliorating UC may be closely related to their ability to inhibit inflammation and maintain intestinal barrier integrity.

Oral administration of NAF AuNRs effectively alleviated DSS-induced weight loss, reduced DAI scores, and improved other inflammation and intestinal barrier-related phenotypes, suggesting their potential role in colitis management. However, several limitations remain in this study. First, a key limitation of the current study lies in its exclusive focus on the T30 nucleic acid sequence, leaving the therapeutic profiles of alternative sequences uncharacterized within the NAF AuNR system. This constraint necessitates systematic comparative analyses to delineate sequence-dependent therapeutic variations. Future investigations should incorporate a broader spectrum of sequence architectures to establish structure–activity relationships, enabling rational selection of optimized nucleic acid motifs for UC intervention. Second, while we have demonstrated that NAF AuNRs can reduce inflammation and restore the intestinal barrier, the precise underlying mechanisms remain unclear and warrant further investigation. Meanwhile, animal identification methods and procedural parameters are potential confounding factors that can influence physiological outcomes [49]. In this study, we minimized the impact of invasive labeling by implementing post-anesthetic tagging protocols, a 7-d acclimatization period, and controlled cohort comparisons to reduce pain, stress, and variability in outcomes. However, future studies should adopt noninvasive biometric tracking systems (e.g., fur pattern recognition) to further eliminate methodological interference and ensure more accurate evaluation of therapeutic effects. Third, longitudinal toxicological assessment constitutes a critical determinant for biomedical implementations of engineered biomaterials. Extant literature substantiates AuNP safety profiles in therapeutic applications (over 6 months) through multicenter trials [50], and NAF AuNRs also exhibited favorable biocompatibility profiles within the current experimental parameters (7-d observation window). Notwithstanding these validations, the intrinsic nondegradability of AuNRs mandates systematic surveillance of persistence-mediated pathophysiological progressive bioaccumulation in organs.

In this study, inspired by characteristics of SNAs, we introduced a novel treatment approach for UC using nucleic acid-modified nanomaterials, NAF AuNRs here, through oral administration (Fig. 1). These NAF AuNRs exhibited simple production and high intestinal stability. The nucleic acid shell coated on the AuNRs strengthened the biocompatibility of AuNRs, also prompting their targeted colitis ability. In vivo experiments demonstrated that NAF AuNR treatment could efficiently alleviate colonic inflammation, restore intestinal barrier function, and modulate gut microbiota, while the molecular mechanisms and long in-depth safety needed to be further explored and advanced in clinical application. Nevertheless, this study offers a new promising therapeutic avenue for UC and reveals more function of nucleic acid-modified nanomaterials, providing novel insights for their application.

## Data Availability

All data used related to the current study are available from the paper, or the Supplementary Materials, or corresponding author on reasonable request.
