# Supplementary material for: Nucleic Acid-Functionalized Gold Nanorods Modulate Inflammation and Dysregulated Intestinal Barriers for Treatment of Ulcerative Colitis
Source: Biomater Res. 2025 Apr 18;29:0195. doi: 10.34133/bmr.0195 (PMC12006742; doi:10.34133/bmr.0195)
Supplement: Supplementary 1 — Figs. S1 to S13 Table S1 [file bmr.0195.f1.docx]

**Nucleic Acid Functionalized Gold Nanorods Modulates Inflammation and Dysregulated Intestinal Barriers for Treatment of Ulcerative Colitis**

Wanghong He^a,1^, Yanxue Wang^a,1^, Yifan Zhao^a^, Bingqing Wu^a^, Yilong Chen^a^, Lu Jia^a^, Xinfeng Tan^b,**^, Yi Liu^a,*^

*^a^ Laboratory of Tissue Regeneration and Immunology and Department of Periodontics, Beijing Key Laboratory of Tooth Regeneration and Function Reconstruction, School of Stomatology, Capital Medical University, Beijing 100050, China*

*^b^ State Key Laboratory of Tribology in Advanced Equipment, Tsinghua University, Beijing 100084, China*

*^*^ Corresponding authors: tanxinfeng@tsinghua.edu.cn; lililiuyi@163.com*

*^1^ These authors contributed equally to this work.*


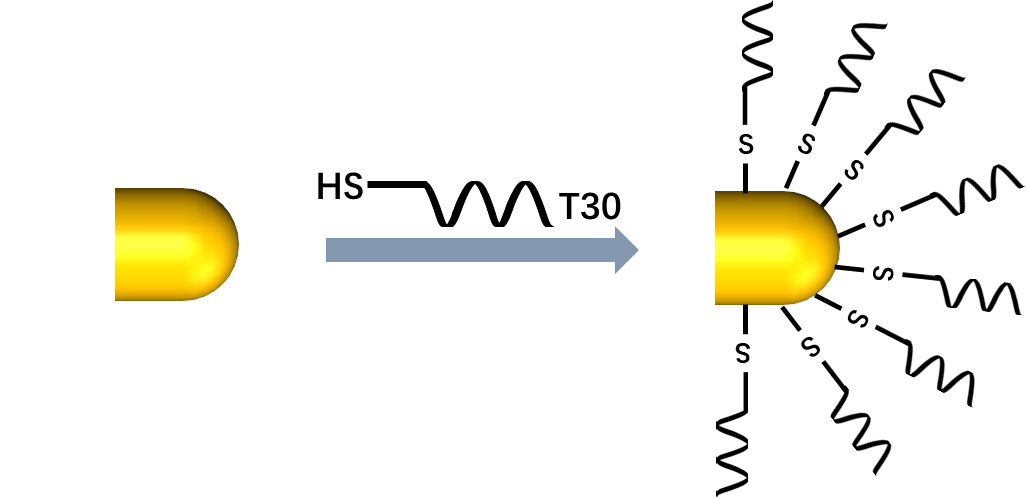


**Figure S1**. Schematic illustration showing the formation of NAF AuNRs.


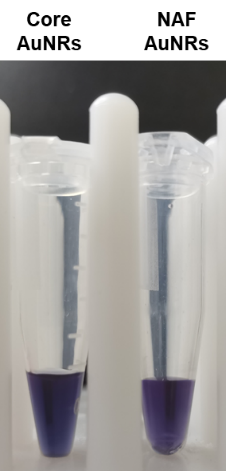


**Figure S2**. Image of core and NAF AuNRs.


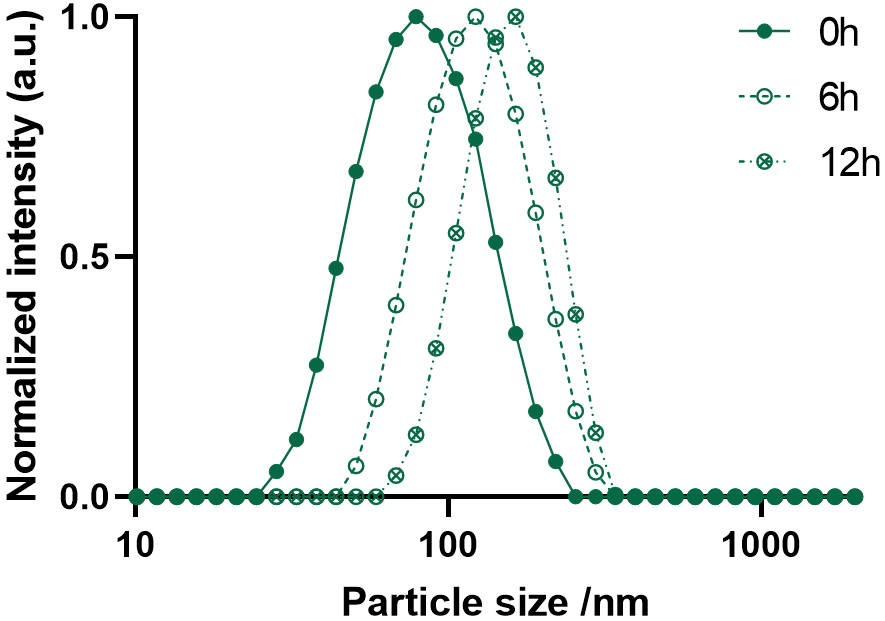


**Figure S3**. Hydrodynamic sizes of NAF AuNRs in artificial gastric fluid (AGF) for 6h and 12h.


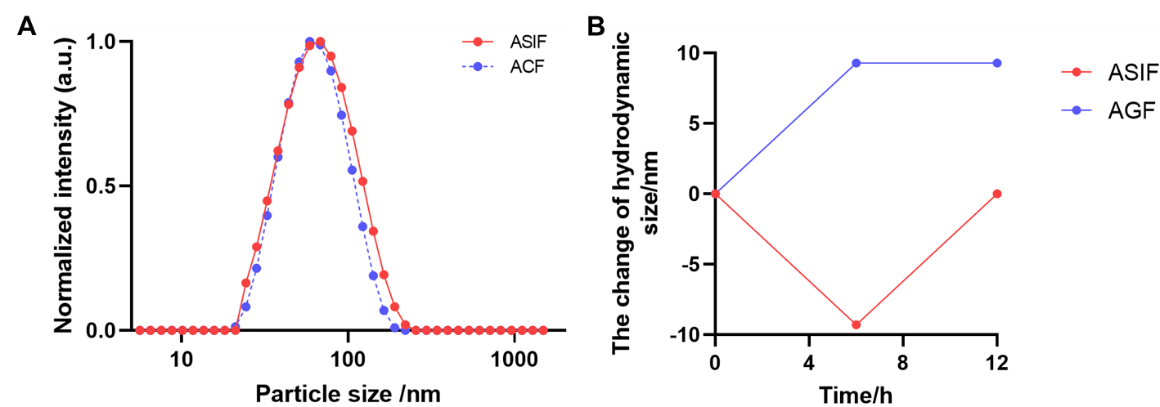


**Figure S4**. Characterization of gastrointestinal stability of NAF AuNRs. A. hydrodynamic sizes of NAF AuNRs in artificial small intestinal fluid (ASIF) and artificial colon fluid (ACF). B. The hydrodynamic size changes of NAF AuNRs in ASIF and ACF for 6h and 12h.


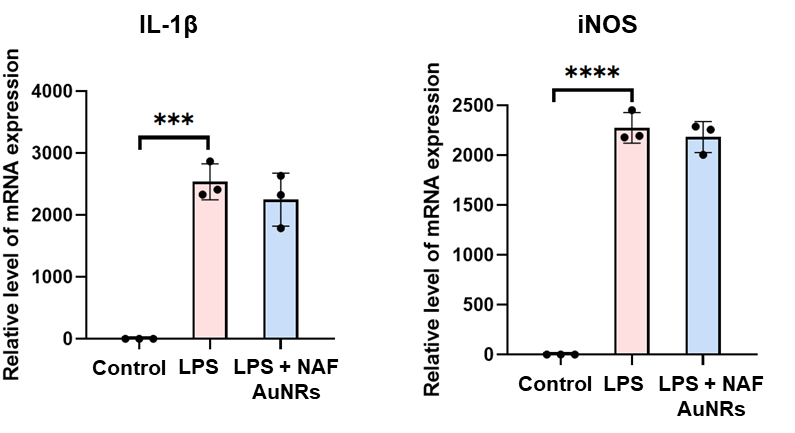


**Figure S5**. Real-time PCR analysis of inflammatory factors of macrophages, including IL-1β and iNOS, on day 1 (effect size of η^2^ = 0.956, 0.991).


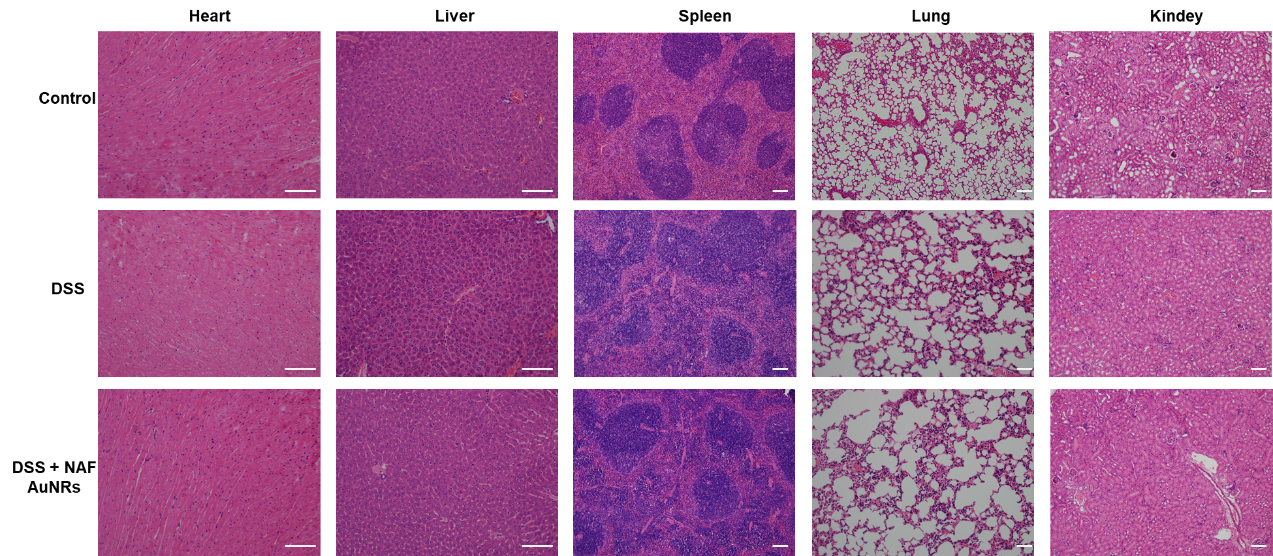


**Figure S6**. H&E staining of the major organs from mice receiving NAF AuNRs treatment. Scale bar: 100 μm.


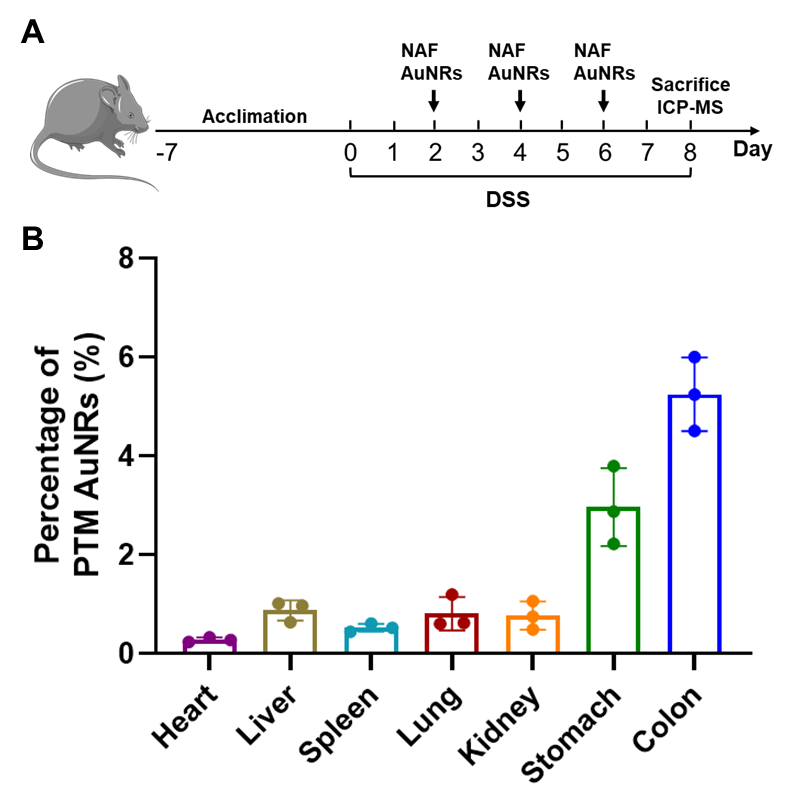


**Figure S7**. Biodistribution of gold accumulation in organs following NAF AuNRs treatment termination. A. Schematic representation of NAF AuNRs treatment and ICP-MS quantification. B. NAF AuNRs content of major organs relative to therapeutic dosage.


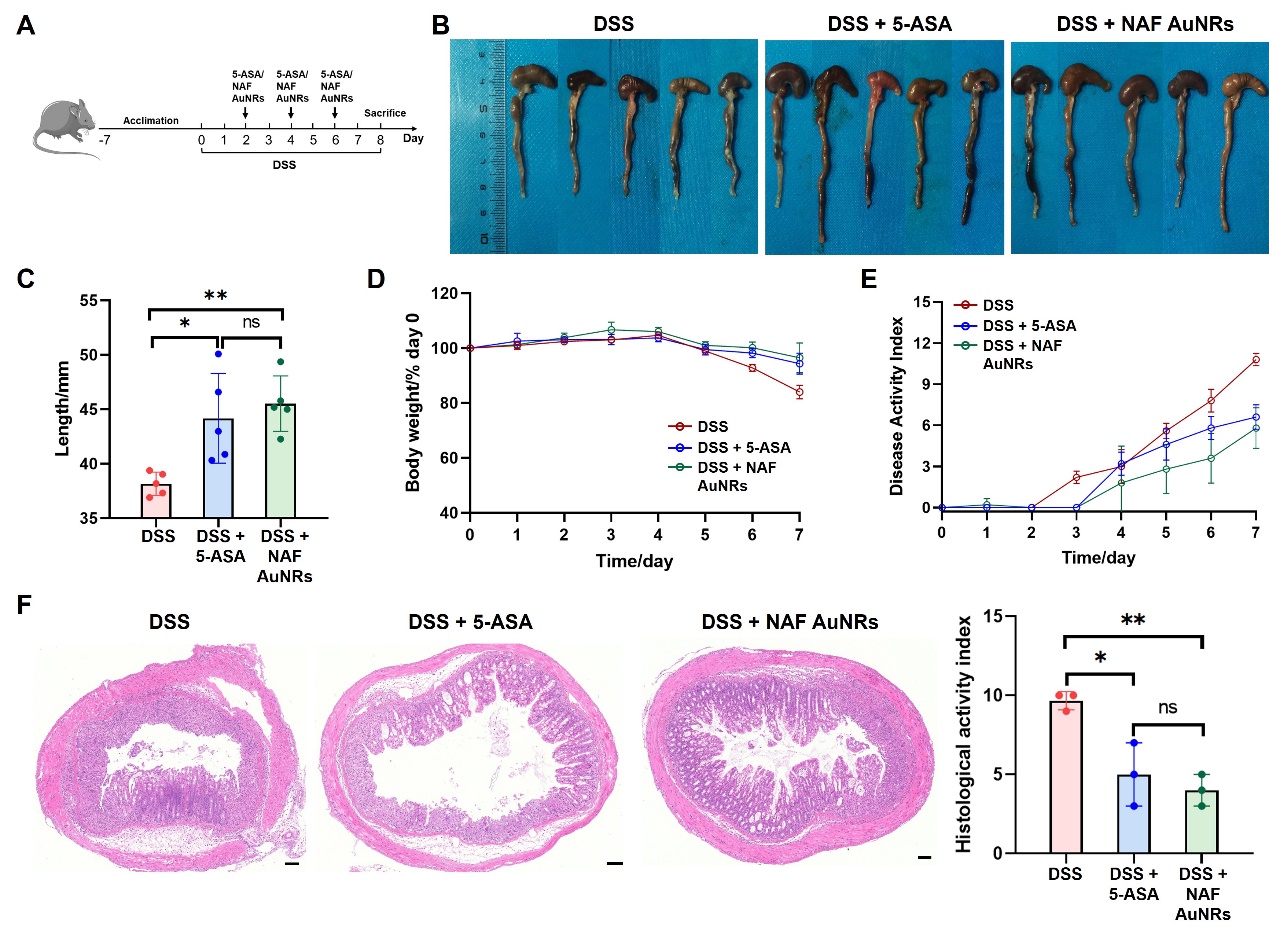


**Figure S8**. Evaluation of the efficacy of 5-ASA and NAF AuNRs treatment in the mice with DSS-induced ulcerative colitis. (A) Schematic illustration of the establishment and therapeutic schedule of the DSS-induced ulcerative colitis mice. (B) Macroscopic colon appearance of mice with different treatments. (C) Colon length of mice with different treatments (effect size of η^2^ = 0.609). (D) Body weight and (E) DAI scores change in each group for 7 days. (F) Representative images of HE staining image and HAI of each group (effect size of η^2^ = 0.837). Scale bar: 100μm. *P < 0.05, **P < 0.01.


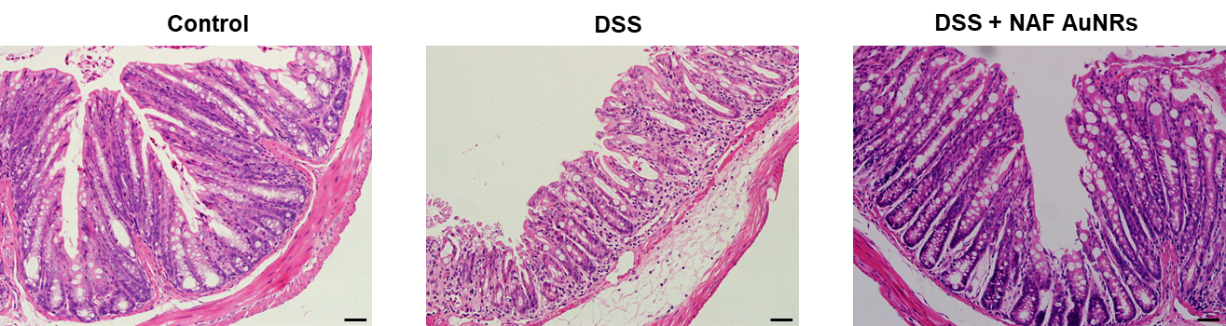


**Figure S9**. H&E staining images of colon tissues. Scale bar: 20 μm.


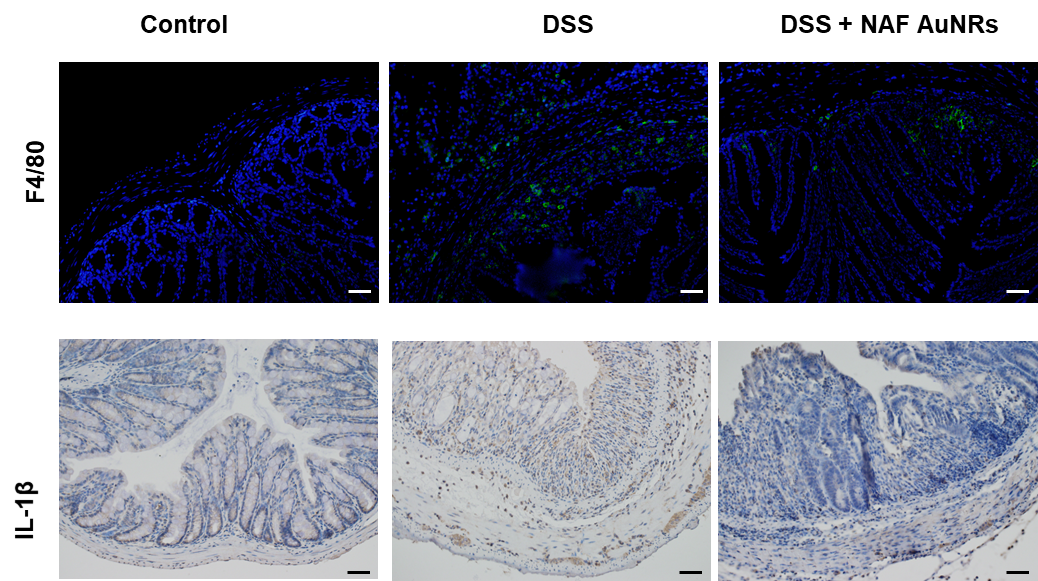


**Figure S10**. Infiltration of macrophage in colon tissue of DSS-induced mice by immunofluorescence method and expression of IL-1β in the colon tissue of DSS-induced mice by immunohistochemistry method. Scale bar: 50 μm.


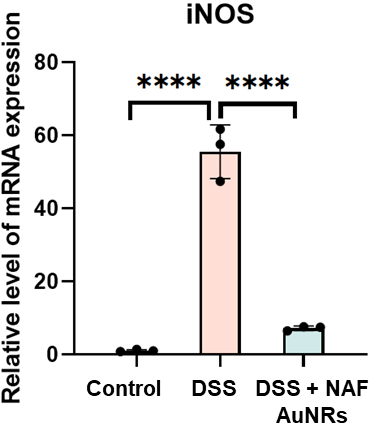


**Figure S11**. Real-time PCR analysis of iNOS of colon tissues of DSS-induced mice on day 8 (effect size of η^2^ = 0.980).


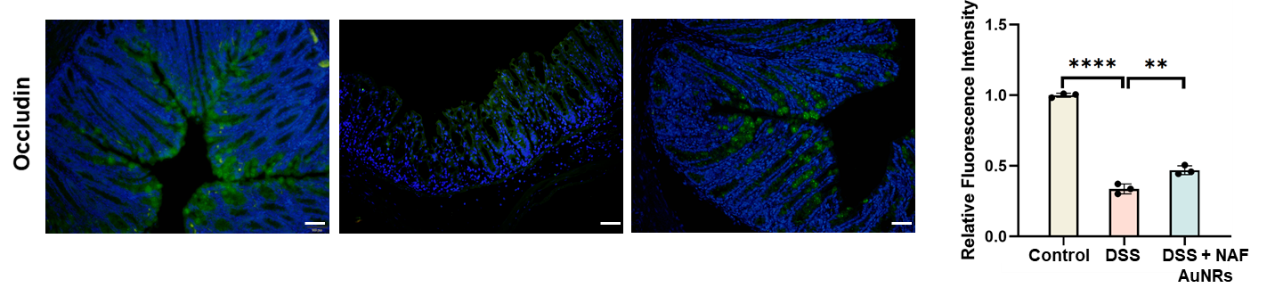


**Figure S12**. Expression of Occludin in the colon tissue of DSS-induced mice by immunofluorescence method (effect size of η^2^ = 0.993). Scale bar: 50 μm.


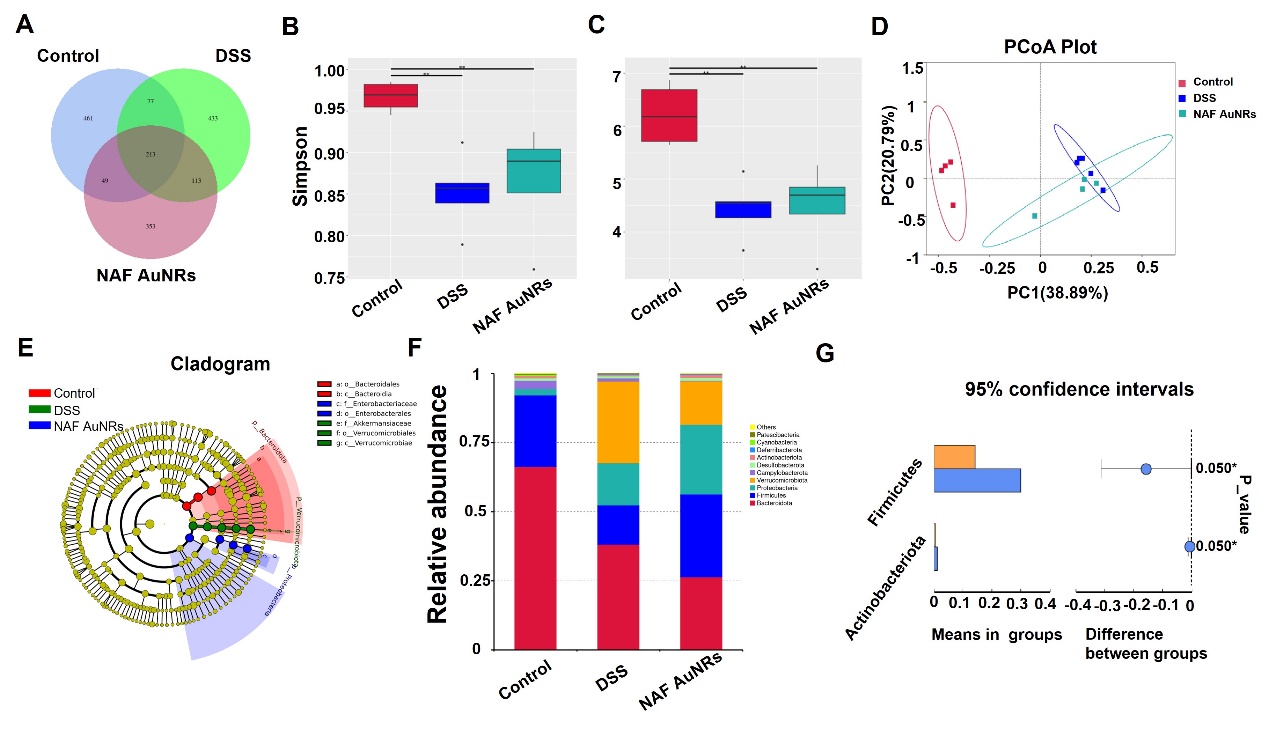


**Figure S13**. Gut microbiota composition modulated by NAF AuNRs in colitis induced by DSS. (A) Venn diagram of shared and unique species in three groups; (B, C) The alpha diversity of gut microbiota, displayed by Simpson and Shannon index; (D) The β-diversity of gut microbiota, displayed by principal coordinate index (PCoA) plot; (E)Microbial biomarkers were assessed by LEfSe in the control, DSS, and NAF AuNRs groups; (F) Distribution of gut microbiota at the phylum level (G) Significant phyla alterations in the DSS group and the NAF AuNRs group.

**Table S1** Primer sequences used in this study.

| Gene | Primer | Sequence (5’-3’) |
| --- | --- | --- |
| *TNF-α* | Forward | CAGGGGCCACCACGCTCTTC |
|  | Reverse | TTTGTGAGTGTGAGGGTCTGG |
| *IL-6* | Forward | CCGGAGAGGAGACTTCACAG |
|  | Reverse | CAGAATTGCCATTGCACAAC |
| *IL-1β* | Forward | TGACGGACCCCAAAAGATGA |
|  | Reverse | TCTCCACAGCCACAATGAGT |
| *iNOS* | Forward | TGACATCGACCAGAAGCTGT |
|  | Reverse | CACTGAGTTCGTCCCCTTCT |
| *Mucin-2* | Forward | ATGCCCACCTCCTCAAAGAC |
|  | Reverse | GTAGTTTCCGTTGGAACAGTGAA |
| *Occludin* | Forward | TGAAAGTCCACCTCCTTACAGA |
|  | Reverse | CCGGATAAAAAGAGTACGCTGG |
| *ZO-1* | Forward | GCTTTAGCGAACAGAAGGAGC |
|  | Reverse | TTCATTTTTCCGAGACTTCACCA |
| *GAPDH* | Forward | TGTAGACCATGTAGTTGAGGTCA |
|  | Reverse | AGGTCGGTGTGAACGGATTTG |
